# Genetic Characteristics of Latvian Patients with Familial Hypercholesterolemia: The First Analysis from Genome-Wide Sequencing

**DOI:** 10.3390/jcm12155160

**Published:** 2023-08-07

**Authors:** Gustavs Latkovskis, Raimonds Rescenko-Krums, Georgijs Nesterovics, Monta Briviba, Vita Saripo, Dainus Gilis, Elizabete Terauda, Ruta Meiere, Gunda Skudrina, Andrejs Erglis, Joana Rita Chora, Mafalda Bourbon, Janis Klovins

**Affiliations:** 1 Institute of Cardiology and Regenerative Medicine, University of Latvia, LV-1004 Riga, Latviavita.saripo@inbox.lv (V.S.); elizabete.terauda@gmail.com (E.T.);; 2 Latvian Center of Cardiology, Pauls Stradins Clinical University Hospital, LV-1002 Riga, Latvia; 3 Faculty of Medicine, University of Latvia, LV-1004 Riga, Latvia; 4 Latvian Biomedical Research and Study Centre, LV-1067 Riga, Latvia; 5 Department of Health Promotion and Prevention of Noncommunicable Diseases, National Institute of Health Dr. Ricardo Jorge, 164-9016 Lisbon, Portugal; 6 Department of Chemistry and Biochemistry, BioISI—BioSystems & Integrative Sciences Institute, Faculty of Sciences, University of Lisbon, 1649-004 Lisbon, Portugal

**Keywords:** familial hypercholesterolemia, low-density lipoprotein cholesterol, genetic study, monogenic, whole-genome sequencing, registry

## Abstract

Background: There is limited data on the genetic characteristics of patients with familial hypercholesterolemia (FH) in Latvia. We aim to describe monogenic variants in patients from the Latvian Registry of FH (LRFH). Methods: Whole genome sequencing with 30× coverage was performed in unrelated index cases from the LRFH and the Genome Database of Latvian Population. *LDLR*, *APOB*, *PCSK9*, *LDLRAP1*, *ABCG5*, *ABCG8*, *LIPA*, *LPA*, *CYP27A1*, and *APOE* genes were analyzed. Only variants annotated as pathogenic (P) or likely pathogenic (LP) using the FH Variant Curation Expert Panel guidelines for *LDLR* and adaptations for *APOB* and *PCSK9* were reported. Results: Among 163 patients, the mean highest documented LDL-cholesterol level was 7.47 ± 1.60 mmol/L, and 79.1% of patients had LDL-cholesterol ≥6.50 mmol/L. A total of 15 P/LP variants were found in 34 patients (diagnostic yield: 20.9%): 14 in the *LDLR* gene and 1 in the *APOB* gene. Additionally, 24, 54, and 13 VUS were detected in *LDLR, APOB,* and *PCSK9*, respectively. No P/LP variants were identified in the other tested genes. Conclusions: Despite the high clinical likelihood of FH, confirmed P/LP variants were detected in only 20.9% of patients in the Latvian cohort when assessed with genome-wide next generation sequencing.

## 1. Introduction

Familial hypercholesterolemia (FH) is an autosomal semidominant inherited disorder characterized by impaired low-density lipoprotein (LDL) hepatic clearance via LDL receptors (LDLR), lifelong high cholesterol levels, and the development of early atherosclerosis [1]. The most common causative genetic mechanisms are loss of function variations of genes coding for *LDLR* (OMIM #606945, #143890) and apolipoprotein B (*APOB*; OMIM #107730, #144010) or gain of function variations of genes coding for proprotein convertase subtilisin/kexin type 9 (*PCSK9*; OMIM #607786, #603776) (2). Homozygous loss of function variants in LDLR adaptor protein-1 (*LDLRAP1*; OMIM #603813, #605747) may also rarely lead to the FH phenotype [2]. Other genetic disorders such as sitosterolemia (*ABCG5*/*ABCG8* genes) or lysosomal acid lipase deficiency (*LIPA* gene) may mimic the FH phenotype but are rarely encountered [3,4].

Genetic testing is an important aspect of the diagnosis and management of FH [1,2]. The presence of a disease-specific pathogenic variant adds significantly to the correct diagnosis, is associated with a 6 to 22 times higher risk of coronary artery disease, and prompts focused genetic cascade screening in first-degree relatives [1,5,6]. The spectrum of causal FH variants varies significantly among populations, which necessitates sequencing of the three candidate genes as a default strategy [7]. Moreover, only pathogenic (P) and likely pathogenic (LP) variants meeting criteria defined by the American College of Medical Genetics and Genomics and the Association for Molecular Pathology (ACMG/AMP) 2015 algorithm should be regarded as causal for FH [8,9]. The Clinical Genome Resource FH Variant Curation Expert Panel has further optimized the existing ACMG/AMP framework of *LDLR* variants for disease-specific classification in FH [10].

The Latvian Registry of Familial Hypercholesterolemia was established in 2015, and we have previously reported the first results of the clinical data [11]. In Latvia, genetic testing is reimbursed only in cases of suspected homozygous FH; thus, the genetic status has been unknown in the vast majority of patients with suspected heterozygous FH. Therefore, the genetic characteristics of FH patients in Latvia remain largely unknown. The aim of this study was, for the first time, to evaluate the prevalence and spectrum of monogenic variants in Latvian FH patients by using whole-genome sequencing.

## 2. Materials and Methods

### 2.1. Study Population

Patients with definite, probable, or possible FH based on Dutch Lipid Clinic Network criteria were included in this study from the Latvian Registry of FH [1]. The Registry was established in February 2015 by the Institute of Cardiology and Regenerative Medicine in collaboration with the Pauls Stradins Clinical University Hospital. The general inclusion criteria and description of the Registry have been previously reported [11]. Secondary causes such as hypothyroidism, nephrotic syndrome, or drug therapies were routinely excluded in this cohort. By the end of 2022, more than 1177 patients have been included in the Registry, among whom there were 141, 301, and 497 probands with respective definite, probable and possible FH, and 81 relatives with clinical FH diagnoses. For this analysis, mostly index cases with clinically definite or probable FH were selected. In total, 192 patients were submitted to the WGS to match the 96 well plate format. Patients were selected from the clinically definite or probable FH groups based on the chronology of registration. Additionally, two index patients with possible FH were included with LDL-cholesterol levels above 6.5 mmol/L. Premature coronary artery disease (CAD) was defined as CAD <55 years in men and <60 years in women. High and very high cardiovascular risk were defined according to the 2021 European Society of Cardiology Guidelines on cardiovascular disease prevention in clinical practice [12].

The study was conducted in collaboration with The Genome Database of Latvian Population (LGDB), which is a government-financed population-based biobank run by the Latvian Biomedical Research and Study Centre (LBMC), ensuring the collection of both blood samples and relevant anthropometric data according to their standard procedures [13]. The study protocol conforms with the ethical guidelines of the 1975 Declaration of Helsinki. All included patients have signed the project-specific written informed consent and additional broad informed consent for participation in the LGDB, both approved by the local medical ethics committee (Approval No. 300115-7L and updated approval No. 131218-24L) for the LRFH and the Central Medical Ethics Committee (approvals No. 01-29.1/2429 and No. 1/19-04-05) for the LGDB [13].

### 2.2. DNA Extraction and Whole-Genome Sequencing

The genomic DNA was extracted from peripheral blood leukocytes using a phenol–chloroform extraction method according to LGDB standard procedures [13]. Further, the PCR-free DNA libraries were prepared using the MGIEasy PCR-Free DNA Library Prep Set (MGI Tech Co., Ltd., Shenzhen, China) on the MGISP-960 High-throughput Automated Sample Preparation System (MGI Tech Co., Ltd., Shenzhen, China) and sequenced on the DNBSEQ-T10×4RS sequencing platform (MGI Tech Co., Ltd., Shenzhen, China) using the DNBSEQ-T10×4RS High-throughput Sequencing Set (FCL PE150) (MGI Tech Co., Ltd., Shenzhen, China), providing at least 150 bp paired-end sequencing reads (30× sequencing coverage) per sample. Out of the 192 samples, 29 did not reach 100 Gb of data, which was our predetermined quality threshold. These samples were therefore excluded from further analysis, resulting in the final total of 163 patients in our study.

### 2.3. Variant Calling and Annotation

Sequences were processed according to GATK best practices. Sequenced reads were trimmed with trim-galore [14] v0.6.7 and aligned to the GRCh38 human reference genome using BWA-mem2 [15] v2.2.1, with mapped read quality assessed using bamQC [16]. Aligned reads were sorted with samtools [17] v1.9, duplicates marked with GATK MarkDuplicatesSpark [18] v4.2.6.1, and Base Quality Score Recalibrated with dbSNP146 using GATK applyBQSR. Variants were then called using GATK HaplotypeCaller with the -ERC GVCF option enabled for further combined variant calling using GATK GenotypeGVCFs on 50 MB chunks. ANNOVAR [19] v2020-06-08 was used to automate annotation and consequence determination with Ensembl v107.5f39899 Variant Effect Predictor (VEP) v107.0 command line tool [20]. Parallel [21] v20220522 was used to distribute computation on Riga Technical University HPC cluster computers, while Singularity [22] was used to install the necessary software.

### 2.4. Data Analysis

For this study, *LDLR*, *APOB*, *PCSK9*, *LDLRAP1*, *ABCG5*, *ABCG8*, *LIPA*, *LPA*, *CYP27A1*, and *APOE* genes were analyzed. Variants were classified as P/LP using the FH Variant Curation Expert Panel (VCEP) guidelines for *LDLR* [10], and adaptations for *APOB* and *PCSK9* were used based on the general guidelines defined by the ACMG/AMP [8].

### 2.5. Statistical Analysis 

Continuous variables were shown as mean arithmetic and standard deviation if normally distributed or as median and interquartile range if the distribution was non-normal. Categorical variables were shown as counts and percentages. Normally distributed continuous variables were compared with the Student’s *t*-test for independent samples for two groups. Non-normally distributed continuous variables were compared with the Mann–Whitney U test. Categorical variables were compared using the Chi–square test or Fisher’s exact test, as appropriate. All results were analyzed using IBM SPSS 29.0 Software. Results with *p* values below 0.05 were considered statistically significant.

## 3. Results 

### 3.1. Clinical Characteristics

The clinical characteristics of the 163 participants included in the study are summarized in Table 1. In brief, the mean age was 52.9 ± 11.4 years, and 67.5% of patients were women (*n* = 110). The clinical DLCN diagnosis was definite, probable, or possible FH in 56 (34.4%), 105 (64.4%), and 2 (1.2%) patients, respectively. The mean highest documented LDL-cholesterol level was 7.47 ± 1.60 mmol/L, and 98.2% of patients had LDL-cholesterol ≥5.00 mmol/L. The mean LDL-cholesterol at the time of inclusion in the registry was 5.43 ± 2.09 mmol/L, and at the latest visit it was 4.30 ± 2.19 mmol/L. Only 14 patients (8.6%) reported that they had been on lipid-lowering therapy when the highest documented LDL-cholesterol levels were recorded. 

### 3.2. Characteristics Based on Genetic Data 

**Figure 1:**
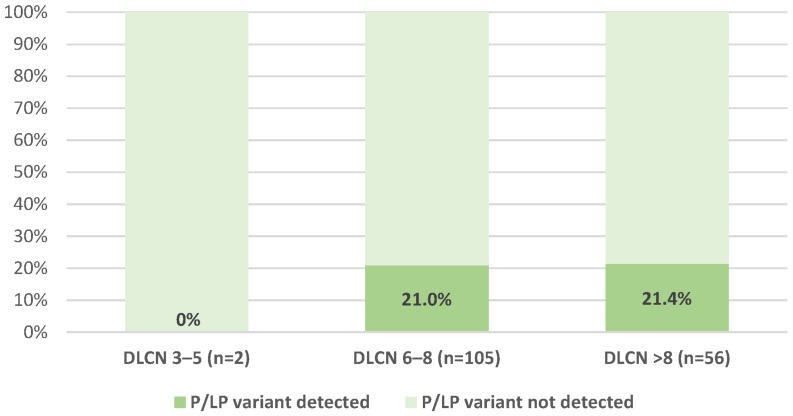
The proportion of detected pathogenic/likely pathogenic variants according to the Dutch Lipid Clinic Network score; Abbreviations: DLCN, Dutch Lipid Clinic Network; LP, likely pathogenic; P, pathogenic.

A total of 15 P/LP variants were found in 34 patients (diagnostic yield 20.9%), 14 in the *LDLR* gene and 1 in the *APOB* gene, all in exons (Table 2, Supplementary Figure S1). Additionally, 24, 54, and 13 VUS (Variants of Uncertain Significance) were also detected in the *LDLR, APOB,* and *PCSK9* genes, respectively (Supplementary Figure S2 and Supplementary Table S1). No P/LP variants were identified in *LDLRAP1*, *ABCG5*, *ABCG8*, *LIPA*, *LPA*, *CYP27A1*, or *APOE* genes. The rate of P/LP variants according to clinical diagnosis subgroups as defined by DLCN criteria is shown in Figure 1 and Table 3. The following P/LP positivity rate among women and men was observed: 24.5% (*n* = 27/110) and 13.2% (*n* = 7/53), respectively (*p* = 0.095).

At all analyzed time points, LDL-cholesterol levels were numerically higher in P/LP patients, but the differences from patients without P/LP variants were not statistically significant (Table 4). The highest mean documented LDL-cholesterol levels were 7.80 ± 1.82 mmol/L and 7.38 ± 1.54 mmol/L in patients with and without P/LP variants, respectively (*p* = 0.176). At baseline, when patients were included in the Registry, the corresponding LDL-cholesterol levels were substantially lower in both groups: 5.65 ± 2.00 and 5.38 ± 2.11 (*p* = 0.490).

When the lowest documented on-treatment LDL-cholesterol levels were compared to the highest measured LDL-cholesterol levels in patients with at least one follow-up visit, patients with P/LP variants tended to have higher levels and less pronounced mean and median percent reductions (Table 4).

The highest documented LDL-cholesterol ≥6.5 mmol/L was recorded in 79.1% (*n* = 129) of patients, but the proportion of patients with this severe hypercholesterolemia was similar in both groups: 82.4% (*n* = 28/34) in patients with P/LP variants and 78.3% (*n* = 101/129) in patients without P/LP variants (*p* = 0.604).

Interestingly, tendon xanthomas were found in 17.6% (*n* = 6/34) of patients with P/LP variants compared to 22.5% (*n* = 29/129) without P/LP variants (*p* = 0.541). Premature arcus cornealis (found at age <45 years) was reported in 8.8% (*n* = 3/34) and 3.1% (*n* = 4/129), respectively (*p* = 0.159).

A history of premature CAD in at least one relative was positive in 35.3% (*n* = 12) of patients with P/LP variants and in 38.8% (*n* = 50) of patients without P/LP variants (*p* = 0.787). Low-density lipoprotein cholesterol levels above the 95th percentile in a first-degree relative were documented in 47.1% (*n* = 16) and 41.9% (*n* = 54) of patients with and without P/LP variants, respectively (*p* = 0.432). Children with LDL-cholesterol levels above the 95th percentile had been registered in 3 cases (2.3%) among patients without P/LP variants and in none among patients with P/LP variants.

Patients with P/LP variants had slightly lower highest-documented TG levels: median (interquartile range, IQR) 1.61 (1.19–2.21) mmol/L compared to 1.79 (1.31–2.30) mmol/L in patients without P/LP variants (*p* = 0.474). Fewer patients with P/LP variants had TG levels ≥2.3 mmol/L (20.6% vs. 22.5%, *p* = 0.839). 

## 4. Discussion

This is the first study to report genome-wide sequencing data on patients with FH in Latvia. In total, a P or LP variant was found in 20.9% of genotyped FH patients. Compared to similar FH studies in other populations where the prevalence of monogenic P or LP variants is, on average, 20 to 40%, our findings fit at the lowest end of the range [23]. A similar diagnostic yield was reported in the UK: 21.3% among 15,688 index cases [24]. However, many authors report a diagnostic yield as high as 50–80% [25,26,27,28]. The positive predictive value of definite FH as defined by Simon Brooms and DLCNB criteria is similar, but varies across populations, from 35 to 37% in Korea to 80–90% in Spanish and English subjects [27]. Although reports published before 2015, when ACMG Guidelines were issued and afterwards adapted for FH, may have overestimated the true prevalence of causal variants the diagnostic yield in recent publications from other populations has been higher than in our study [8,10,29].

Several factors could be considered as potential explanations for the lower prevalence of P/LP variants in our cohort. One possibility is that the Latvian genetic variant spectrum is rather different from most previously reported populations that are geographically and therefore likely genetically distant from the Latvian population.

Alternative molecular etiologies and phenocopies should always be considered in patients without P/LP variants [7]. The advantage of this study was that GWS allowed us to evaluate P/LP variants in other genes such as *APOE*, *LPA*, *CYP27A1*, *LIPA,* and *ABCG5/ABCG8*. Although no P/LP variants were found in these genes, we cannot exclude that some of the identified VUS in the three major candidate genes or other genes may be clinically significant. The probability of other monogenic causes of the FH phenotype in P/LP variant-negative patients is usually regarded as very low, and unexplained cases are likely to be rather heterogeneous [30,31].

One reason for our low positive rate is that Multiplex Ligation-dependent Probe Amplification (MLPA) was not used to detect larger gene rearrangements. Although MLPA has been a commonly used method to diagnose copy number variations (CNVs) in the 2000s, in the latter years, whole genome NGS has been promising as an alternative to identify them [32,33]. We cannot, however, exclude that with the NGS analysis approach described in this study, we may have missed genetic variants such as deletions, duplications, translocations, and reverse or complex duplications >50 bp in length [33]. It has been recommended to perform *LDLR* deletion/duplication analysis in routine genetic testing for FH [7]. In our study population, all 14 identified P/LP variants were point substitutions. In the overview of the ClinVar database by the FH Variant Curation Expert Panel, the reported CNV rate was around 6% of the total number of *LDLR* FH-associated variants [29]. In our small sample, one may expect around two to three CNV cases, which could also be none due to a play of chance. Therefore, undiagnosed CNVs can explain a small fraction of the severe phenotypes in our cohort. Also, we found 24 VUS in LDLR in our studied patients. These variants, after functional studies and other evidence are collected, can be the cause of disease and would therefore increase our positivity rate.

Polygenic hypercholesterolemia may be an explanation for the severe hypercholesterolemia in a substantial number of patients without P/LP variants. Natarajan et al. have demonstrated that among patients with severe hypercholesterolemia, polygenic hypercholesterolemia, as assessed by the top 5th percentile of a 2 million SNP score, was ten times more prevalent (23%) than monogenic FH (2%) in samples from six cohorts of general populations [34]. Other authors, however, have reported that the mean polygenic risk scores for LDL-cholesterol among FH mutation-negative patients are only modestly higher than in FH mutation-positive cases, and therefore the overall impact of polygenic mechanisms in mutation-negative individuals with high LDL-cholesterol is likely low [35]. The absolute difference in LDL-cholesterol levels between the highest and lowest polygenic risk score (PRS) deciles may be as high as 1.1 mmol/L [36]. It should also be noted that the prevalence of polygenic hypercholesterolemia may vary among the studies due to variable score definitions (top 5th, 10th, or 20th percentile of PRS) and populations studied [34,36,37]. In our sample, the majority (79%) of patients without P/LP variants had a severe phenotype with LDL-cholesterol levels ≥6.5 mmol/L, which in our view implies that even if there was a high PRS in these patients, their LDL-cholesterol levels unattributed to polygenic mechanisms would still be well above 5.0 mmol/L, and therefore other mechanisms should be further investigated irrespective of polygenic risk scores. The role of polygenic hypercholesterolemia in the Latvian cohort of FH is currently being studied in an ongoing research project.

We also cannot exclude the possibility that some patients without P/LP variants may have familial combined hyperlipidemia. Indeed, these patients tended to have higher median triglyceride (TG) levels, and there were slightly more patients with TG > 2.3 mmol/L in this subgroup. The caveat of this analysis, however, is that we do not have full data on whether these TG levels were measured in a fasting state in all patients.

Interestingly, in our study, patients with P/LP variants had higher but not significantly higher LDL-cholesterol levels at all investigated time points as compared to patients without P/LP variants. The difference was indeed modest: the mean highest documented LDL-cholesterol level was only 0.32 mmol/L higher in patients with P/LP variants. This observation, in our view, also suggests that among patients without P/LP variants, other causes for a monogenic condition should be suspected.

Another factor leading to a lower diagnostic yield would be that all patients in our cohort were unrelated index cases, and their relatives were excluded. In some of the other studies, relatives were also included in the analyses, which, in our opinion, may increase the positivity rate, but cannot fully explain the difference in overall diagnostic yield. For example, 9 (9.8%) out of 92 patients with P/LP variants (4.4% of the whole study population) were related in the German cohort [25].

Compared to other studied populations, LDL-cholesterol levels in our sample were similar or even higher. For instance, in an Italian cohort, the median untreated LDL-cholesterol was 6.69 mmol/L (258.5 mg/dL) in patients with P/LP variants compared to the median 7.42 mmol/L in our cohort, and the untreated median LDL was 5.35 mmol/L (207 mg/dL) in patients without P/LP variants compared to 7.00 mmol/L in our cohort [26]. Thus, both patients with and without P/LP variants in the Latvian sample had substantially higher LDL-cholesterol levels, but nevertheless, the diagnostic yield was 56.5% in the Italian cohort [26]. Among more than one thousand Czech FH patients, the mean baseline LDL-cholesterol was 6.49 mmol/L, which again was lower as compared to 7.47 mmol/L in our study [38]. In some other studies, we failed to find the mean LDL-cholesterol levels of the study group, which makes it more difficult to compare them with our cohort [25]. Also, we are not certain about several other studies if the baseline reported LDL-cholesterol was the highest ever documented, as in our study or at the time of entry in the cohort, or what the share of treated patients was.

Furthermore, in the Copenhagen General Population Study, a much lower LDL-cholesterol concentration (4.4 mmol/L) was identified as the most optimal threshold with the best specificity and sensitivity to discriminate between P/LP variant carriers and noncarriers for all ages in the general population, but particularly in younger patients aged <40 years [39]. Most of our patients in the study had LDL-cholesterol levels well above 4.4 mmol/L, and with a mean LDL-cholesterol level of 7.51 mmol/L, we believe this cohort well represented a group with severe hypercholesterolemia with a high clinical likelihood of FH. Therefore, we do not believe that our patient cohort would represent a poorly selected sample. Of note, we reported the highest documented LDL-cholesterol as a baseline and not at the time of inclusion in the registry, when patients were first seen in the registry, when LDL-cholesterol levels may have been lower due to more common LLM. In some reports, it was not clearly stated what the baseline values were.

Fourteen patients reported that they were on an LLM when the highest documented LDL-cholesterol was recorded. A caveat of our study was that we did not recalculate hypothetical pretreatment LDL-cholesterol levels in these patients for several reasons. First, in some of the patients, the exact treatment and dose were not precisely known. Second, in some cases, LDL-cholesterol levels were very high despite the alleged treatment, and there were doubts about the credibility of the information or the adherence. In our opinion, these inaccuracies had little impact on the study findings, and if anything, the pretreatment cholesterol values would have been even higher.

The general population in Latvia is known to be at very high cardiovascular risk [12], and the mean non-HDL-cholesterol levels are well above the median in the world [40]. Unhealthy lifestyles, more common premature CAD, and higher LDL-cholesterol levels may therefore all contribute to a slight overdiagnosis of FH in the Latvian population when clinical DLCN criteria are applied, especially when compared to lower-risk countries such as Italy or Denmark.

We did not use Sanger sequencing to confirm the identified P/LP variants in this study. In our view, it is not a major caveat, as recent evidence showed that the NGS approach provides high-quality data that does not require Sanger validation [41].

## 5. Conclusions

Despite the high clinical likelihood of FH, confirmed P/LP variants associated with FH were detected in only 20.9% of patients when assessed with genome-wide next-generation sequencing. No P/LP variants in phenocopy genes were detected in this analysis. Future studies will extend to further analysis of VUS based on VCEP criteria, the search for CNVs, the impact of polygenic mechanisms, and WGS in a larger Latvian sample.

## Figures and Tables

**Table 1:** General characteristics of the study population.

	All Subjects (*n* = 163)
Men ^a^	53 (32.5%)
Age, years ^b^	52.91 ± 11.40
High risk ^c^	24 (14.7%)
Very high risk ^d^	139 (85.3%)
Total cholesterol (highest documented, mmol/L) ^a^	9.82 ± 1.92
LDL-cholesterol (mmol/L) ^b^	
Highest documented	7.47 ± 1.60
At inclusion in the Registry	5.43 ± 2.09
Lowest on-treatment ^e^	3.30 ± 1.55
At the latest follow-up	4.30 ± 2.19
Triglycerides (highest documented, mmol/) ^f^	1.78 (1.28–2.29)
HDL-cholesterol (lowest documented, mmol/L) ^b^	1.48 ± 0.38
LLM at the latest follow-up	
Statin ^a^	116 (71.2%)
High-intensity statin ^a^	83 (50.9%)
Ezetimibe ^a^	49 (30.1%)
PCSK9 inhibitor ^a^	6 (3.7%)
FH diagnosis (based on DLCN criteria) ^g^	
Definite FH ^a^	56 (34.4%)
Probable FH ^a^	105 (64.4%)
Possible FH ^a^	2 (1.2%)
CAD ^a^	102 (62.6%)
Premature CAD ^a^	74 (45.4%)
Arterial hypertension ^a^	59 (36.2%)
Diabetes mellitus ^a^	8 (4.9%)
Type 1 ^a^	1 (0.6%)
Type 2 ^a^	7 (4.3%)
BMI (kg/m^2^) ^b^	26.99 ± 4.11
Obesity ^a,h^	32 (19.6%)
Smoking status	
Current or ex-smokers ^a^	59 (36.2%)
Non-smokers ^a^	104 (63.8%)
Tendon xanthomas ^a^	35 (21.5%)
Corneal arcus before age 45 ^a^	7 (4.3%)
Xanthelasms ^a^	9 (5.5%)
Family history of premature ASCVD ^a^	62 (38.0%)

Abbreviations: ASCVD, atherosclerotic cardiovascular disease; BMI, body mass index; CAD, coronary artery disease; DLCN, Dutch Lipid Clinic Network; FH, familial hypercholesterolemia; HDL, high-density lipoprotein; LDL, low-density lipoprotein; LLM, lipid-lowering medication. ^a^ *n* (%); ^b^ Mean (standard deviation); ^c^ All FH patients with no signs of very-high risk were regarded as high-risk individuals; ^d^ As defined by the 2021 ESC Guidelines on cardiovascular disease prevention. [12]; ^e^ Only for patients who have received LLM at any visit (*n* = 127); ^f^ Median, interquartile range; ^g^ Based on pre-test data before genetic analysis; ^h^ BMI ≥ 30 kg/m^2^.

**Table 2:** List of pathogenic/likely pathogenic variants identified in 34 patients.

Number	Gene	Variant	rsID	Clinvar ID	Classification (Chora et al., 2022) [10]	Heterozygous or Homozygous, VEP Consequence	Number of Cases
1	*LDLR*	g.11089559G>A	rs201016593	250973	Pathogenic	Heterozygous, stop gained	1
		c.11G>A					
		(p.Trp4*)					
2	*LDLR*	g.11105333T>A	rs875989901,	920596	Likely pathogenic	Heterozygous, missense variant	1
		c.427T>A					
		(p.Cys143Ser)					
3	*LDLR*	g.11105436C>T	rs121908026	3686	Pathogenic	Heterozygous, missense variant	2
		c.530C>T					
		(p.Ser177Leu)					
4	*LDLR*	g.11105572C>A	rs756613387	251364	Pathogenic	Heterozygous, stop gained	1
		c.666C>A					
		(p.Cys222*)					
5	*LDLR*	g.11106668T>A	rs139043155	161287	Pathogenic	Heterozygous, missense variant	1
		c.798T>A					
		(p.Asp266Glu)					
6	*LDLR*	g.11107484G>A	rs121908030	3692	Pathogenic	Heterozygous, missense variant	2
		c.910G>A (p.Asp304Asn)					
7	*LDLR*	g.11110697G>A	rs761954844	226344	Pathogenic	Heterozygous, missense variant	6
		c.986G>A					
		(p.Cys329Tyr)					
8	*LDLR*	g.11113313G>A	rs137943601	36453	Likely pathogenic	Heterozygous, missense variant	1
		c.1222G>A (p.Glu408Lys)					
9	*LDLR*	g.11113376G>A	rs28942078	3694	Pathogenic	Heterozygous, missense variant	2
		c.1285G>A					
		(p.Val429Met)					
10	*LDLR*	g.11116928G>A	rs137929307	161271	Pathogenic	Heterozygous, missense variant	1
		c.1775G>A (p.Gly592Glu)					
11	*LDLR*	g.11120224C>T	rs193922569	36458	Likely pathogenic	Heterozygous, stop gained	1
		c.1978C>T					
		(p.Gln660*)					
12	*LDLR*	g.11120380G>A	rs752935814	252161	Pathogenic	Heterozygous, stop gained	3
		c.1998G>A					
		(p.Trp666*)					
13	*LDLR*	g.11105531T>G	rs1600711065	684864	Likely pathogenic	Heterozygous, missense variant	1
		c.625T>G					
		(p.Cys209Gly)					
14	*LDLR*	g.11113383C>T	NA	CA404084995	Likely pathogenic	Heterozygous, missense variant	2
		c.1292C>T					
		(p.Ala431Val)					
15	*APOB*	g.21006288C>T	rs5742904	17890	Pathogenic ^a^	Heterozygous, missense variant	9
		c.10580G>A					
		(p.Arg3527Gln)					

Abbreviations: VEP, Variant Effect Predictor; ^a^ Classified with adaptations of the general guidelines defined by the American College of Medical Genetics and Genomics and the Association for Molecular Pathology [8].

**Table 3:** Prevalence of P/LP variants in *LDLR* and *APOB* genes by FH diagnosis.

Gene with P/LP Variant	DLCN Group		
	Possible (*n* = 2)	Probable (*n* = 105)	Definite (*n* = 56)
*LDLR*	0 (0%)	17 (16.2%)	8 (14.3%)
*APOB*	0 (0%)	5 (4.8%)	4 (7.1%)

Abbreviations: DLCN—Dutch Lipid Clinic Network; LP, likely pathogenic; P, pathogenic.

**Table 4:** Comparison of LDL-cholesterol values and percent reduction in patients with and without P/LP variants.

LDL-Cholesterol		With P/LP Variants	Without P/LP Variants	*p* Value
Highest documented, mmol/L (*n* = 163)	Mean ± SD Median (IQR)	7.80 ± 1.81 7.42 (6.60–8.52)	7.38 ± 1.54 7.00 (6.52–7.98)	0.176 0.234
Baseline at enrollment in the Registry, mmol/L (*n* = 163)	Mean ± SD	5.65 ± 2.00	5.38 ± 2.11	0.490
	Median (IQR)	5.46 (4.07–7.37)	5.30 (3.74–6.72)	0.472
Lowest/best on treatment, mmol/L (*n* = 127) ^a^		(*n* = 25)	(*n* = 102)	
	Mean ± SD	3.64 ± 1.54	3.21 ± 1.55	0.219
	Median (IQR)	3.39 (2.59–4.20)	2.91 (1.97–4.21)	0.158
Latest documented at follow-up, mmol/L (*n* = 163)	Mean ± SD	4.77 ± 2.10	4.18 ± 2.20	0.162
	Median (IQR)	4.40 (2.99–5.92)	3.91 (2.37–5.73)	0.102
Percent reduction from highest documented LDL-cholesterol to lowest/best on treatment in patients with at least one follow-up visit (*n* = 84) ^b^		(*n* = 17)	(*n* = 67)	
	Mean ± SD	54.91 ± 20.13	63.37 ± 14.48	0.051
	Median (IQR)	56.37 (48.45–67.80)	66.47 (53.60–75.13)	0.080

Abbreviations: IQR, interquartile range; LDL, low-density lipoprotein; LP, likely pathogenic; P, pathogenic; SD, standard deviation ^a^ Only patients who have been on LLM at any visit; ^b^ Only patients on lipid lowering medications with a follow-up.

## Data Availability

The data presented in this study are available on request from the corresponding author.

## Supplementary Materials

The following supporting information can be downloaded at: https://www.mdpi.com/article/10.3390/jcm12155160/s1, Figure S1. Variant Effect Predictor annotated consequence of detected FH P/LP variants; Figure S2. Variant Effect Predictor annotated consequence of detected FH VUS variants, defined with CLNSIG value of Conflicting_interpretations_of_pathogenicity and Uncertain_significance; Table S1. Variant Effect Predictor annotated consequence of 91 detected FH VUS variants, defined with CLNSIG value of Conflicting_interpretations_of_pathogenicity and Uncertain_significance.

## Abbreviations

*ABCG5*	gene encoding ATP-binding cassette sub-family G member 5
*ABCG8*	gene encoding ATP-binding cassette sub-family G member 8
ACMG	the American College of Medical Genetics and Genomics
AMP	the Association for Molecular Pathology
*APOB*	gene encoding Apolipoprotein B
*APOE*	gene encoding Apolipoprotein E
CAD	coronary artery disease
CNV	copy number variation
*CYP27A1*	gene encoding cytochrome P450 family 27 subfamily A member 1 or sterol 27-hydroxylase
DLCN	Dutch Lipid Clinic Network
DNA	deoxyribonucleic acid
FH	familial hypercholesterolemia
HDL	high-density lipoprotein
IQR	interquartile range
LDL	low-density lipoprotein
*LDLR*	gene encoding low-density lipoprotein receptor
*LDLRAP1*	gene encoding low-density lipoprotein receptor adaptor protein 1
LGDB	the Genome Database of Latvian Population
*LIPA*	gene encoding lipase A, the lysosomal acid lipase
LBMC	the Latvian Biomedical Research and Study Centre
LLM	lipid lowering medication
LP	likely pathogenic
*LPA*	gene encoding lipoprotein(a)
LRFH	Latvian Registry of Familial Hypercholesterolemia
MLPA	Multiplex Ligation-dependent Probe Amplification
P	pathogenic
*PCSK9*	gene encoding Proprotein convertase subtilisin/kexin type 9
PRS	polygenic risk score
SD	standard deviation
TG	triglycerides
VCEP	Variant Curation Expert Panel
VEP	Variant Effect Predictor
VUS	Variant of Uncertain Significance
