# Supplementary material for: Genetic Characteristics of Latvian Patients with Familial Hypercholesterolemia: The First Analysis from Genome-Wide Sequencing
Source: J Clin Med. 2023 Aug 7;12(15):5160. doi: 10.3390/jcm12155160 (PMC10419451; doi:10.3390/jcm12155160)
Supplement: Supplementary file 1 [file jcm-12-05160-s001.zip › jcm-2503183-supplementary.pdf]

Supplementary Materials

Supplementary Figure S1. Variant Effect Predictor annotated consequence of detected FH P/LP variants.

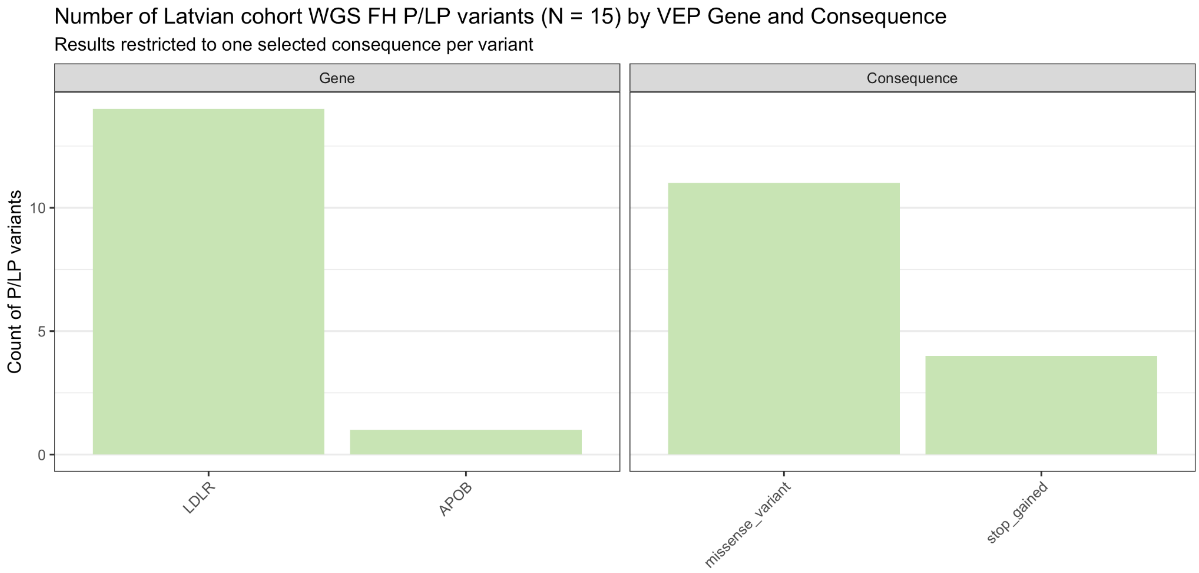

Abbreviations: FH, familial hypercholesterolemia; LP, likely pathogenic; P, pathogenic; VEP, Variant Effect Predictor; WGS, whole genome sequencing.

Supplementary Figure S2. Variant Effect Predictor annotated consequence of detected FH VUS variants, defined with CLNSIG value of Conflicting_interpretations_of_pathogenicity and Uncertain_significance.

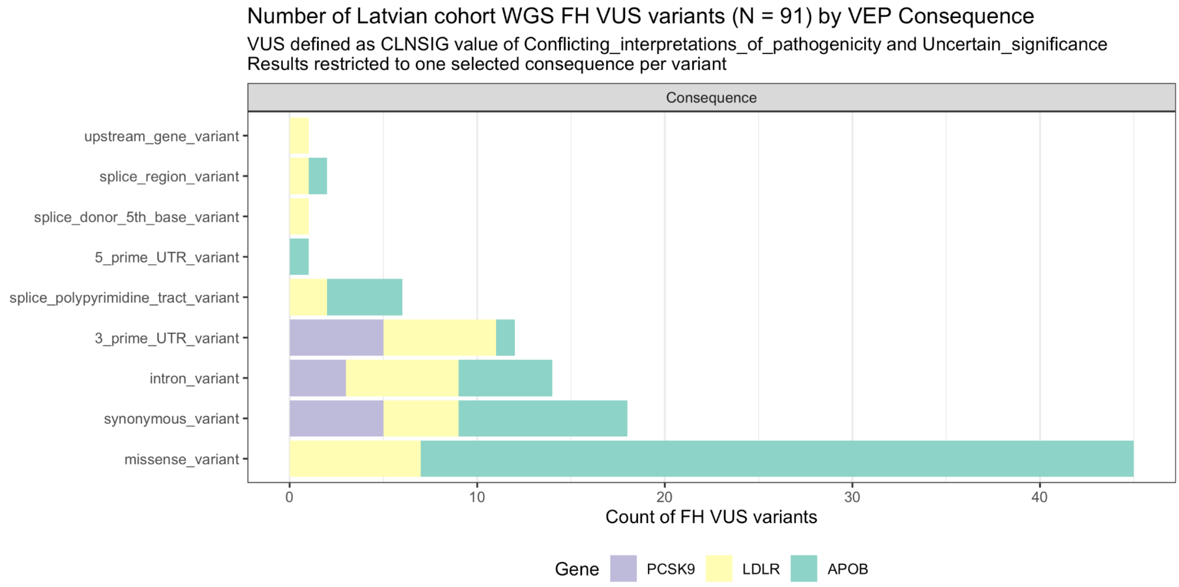

Abbreviations: CLNSIG - clinical significance

Supplementary Table S1. Variant Effect Predictor annotated consequence of 91 detected FH VUS variants, defined with CLNSIG value of Conflicting_interpretations_of_pathogenicity and Uncertain_significance.

SNP Chr_Start_Ref_Alt	rsID	ClinVar ID	Gene	Consequence	Clinical significance
chr1_55039978_C_T	rs28385701	262902	PCSK9	synonymous_variant	Conflicting_interpretations_of_pathogenicity
chr1_55052420_G_A	rs11800243	262906	PCSK9	intron_variant	Conflicting_interpretations_of_pathogenicity
chr1_55052614_G_A	rs11800265	265930	PCSK9	intron_variant	Uncertain_significance
chr1_55052697_C_T	rs7552471	265932	PCSK9	synonymous_variant	Conflicting_interpretations_of_pathogenicity
chr1_55052712_C_T	rs41297883	242028	PCSK9	synonymous_variant	Conflicting_interpretations_of_pathogenicity
chr1_55056233_A_G	rs67578331	265936	PCSK9	intron_variant	Uncertain_significance
chr1_55057360_A_G	rs509504	262899	PCSK9	synonymous_variant	Conflicting_interpretations_of_pathogenicity
chr1_55058082_C_T	rs146924245	413314	PCSK9	synonymous_variant	Conflicting_interpretations_of_pathogenicity
chr1_55063612_G_A	rs189293781	297710	PCSK9	3_prime_UTR_variant	Uncertain_significance
chr1_55063659_C_T	rs28362287	265950	PCSK9	3_prime_UTR_variant	Conflicting_interpretations_of_pathogenicity
chr1_55063755_C_T	rs557622245	297716	PCSK9	3_prime_UTR_variant	Conflicting_interpretations_of_pathogenicity

chr1_55064534_C_T	rs72646537	297741	PCSK9	3_prime_UTR_variant	Uncertain_significance
chr1_55064636_C_T	rs149837083	297742	PCSK9	3_prime_UTR_variant	Uncertain_significance
chr19_11089429_C_T	rs875989886	226299	LDLR	upstream_gene_ variant	Conflicting_interpretations_ of_pathogenicity
chr19_11100340_C_T	rs376207800	161273	LDLR	missense_variant	Conflicting_interpretations_ of_pathogenicity
chr19_11105413_C_T	rs146354103	36464	LDLR	synonymous_variant	Conflicting_interpretations_ of_pathogenicity
chr19_11106668_T_A	rs139043155	161287	LDLR	missense_variant	Conflicting_interpretations_ of_pathogenicity
chr19_11110613_C_T	rs55792959	251548	LDLR	intron_variant	Conflicting_interpretations_ of_pathogenicity
chr19_11110697_G_A	rs761954844	226344	LDLR	missense_variant	Conflicting_interpretations_ of_pathogenicity
chr19_11111506_T_C	rs72658861	36451	LDLR	splice_region_variant, splice_polypyrimidine _tract_variant,intron_ variant	Conflicting_interpretations_ of_pathogenicity
chr19_11113285_C_T	rs13306498	224620	LDLR	synonymous_variant	Conflicting_interpretations_ of_pathogenicity
chr19_11113365_A_T	rs879254850	251762	LDLR	missense_variant	Conflicting_interpretations_ of_pathogenicity
chr19_11113368_T_C	rs879254851	251763	LDLR	missense_variant	Conflicting_interpretations_ of_pathogenicity

chr19_11113679_G_A	rs368889457	251876	LDLR	synonymous_variant	Conflicting_interpretations_of_pathogenicity
chr19_11113778_G_A	rs114891301	431533	LDLR	intron_variant	Conflicting_interpretations_of_pathogenicity
chr19_11116849_G_A	rs17248882	226368	LDLR	splice_polypyrimidine_tract_variant,intron_variant	Conflicting_interpretations_of_pathogenicity
chr19_11120166_C_T	rs5926	237869	LDLR	synonymous_variant	Conflicting_interpretations_of_pathogenicity
chr19_11120527_G_A	rs72658867	36460	LDLR	splice_donor_5th_base_variant,intron_variant	Conflicting_interpretations_of_pathogenicity
chr19_11123210_C_T	rs45508991	36461	LDLR	missense_variant	Conflicting_interpretations_of_pathogenicity
chr19_11128020_T_C	rs780300776	440691	LDLR	missense_variant	Conflicting_interpretations_of_pathogenicity
chr19_11128126_C_A	rs72658868	252310	LDLR	intron_variant	Conflicting_interpretations_of_pathogenicity
chr19_11131335_G_A	rs56270417	251525	LDLR	3_prime_UTR_variant	Conflicting_interpretations_of_pathogenicity
chr19_11132572_C_T	rs886054172	328090	LDLR	3_prime_UTR_variant	Uncertain_significance
chr19_11132685_C_T	rs191796971	891604	LDLR	3_prime_UTR_variant	Uncertain_significance
chr19_11133109_A_C	rs886054176	328109	LDLR	3_prime_UTR_variant	Uncertain_significance
chr19_11133448_G_A	rs184187776	890105	LDLR	3_prime_UTR_variant	Uncertain_significance

chr19_11133526_T_C	rs73925118	890680	LDLR	3_prime_UTR_variant	Uncertain_significance
chr2_21001551_G_A	rs142151703	334060	APOB	3_prime_UTR_variant	Conflicting_interpretations_of_pathogenicity
chr2_21001742_A_G	rs72654427	477801	APOB	synonymous_variant	Conflicting_interpretations_of_pathogenicity
chr2_21001971_G_A	rs12713450	255978	APOB	missense_variant	Conflicting_interpretations_of_pathogenicity
chr2_21001981_C_T	rs1801695	128419	APOB	missense_variant	Conflicting_interpretations_of_pathogenicity
chr2_21002120_G_A	rs144040999	334070	APOB	synonymous_variant	Conflicting_interpretations_of_pathogenicity
chr2_21002247_C_T	rs777718986	373587	APOB	missense_variant	Conflicting_interpretations_of_pathogenicity
chr2_21002400_A_T	rs144018015	627769	APOB	missense_variant	Uncertain_significance
chr2_21002482_T_C	rs72654423	237739	APOB	missense_variant	Conflicting_interpretations_of_pathogenicity
chr2_21002628_A_G	rs61743502	237738	APOB	missense_variant	Conflicting_interpretations_of_pathogenicity
chr2_21002841_A_G	rs570782024	627659	APOB	missense_variant	Conflicting_interpretations_of_pathogenicity
chr2_21003040_C_T	rs1801703	384812	APOB	missense_variant	Conflicting_interpretations_of_pathogenicity
chr2_21003285_C_T	rs149273387	334082	APOB	missense_variant	Conflicting_interpretations_of_pathogenicity

chr2_21004445_C_T	rs373477107	334088	APOB	missense_variant	Conflicting_interpretations_of_pathogenicity
chr2_21004467_A_T	rs370325726	334089	APOB	splice_polypyrimidine_tract_variant,intron_variant	Conflicting_interpretations_of_pathogenicity
chr2_21004631_T_C	rs1801698	334091	APOB	missense_variant	Conflicting_interpretations_of_pathogenicity
chr2_21005391_G_A	rs61744153	237735	APOB	missense_variant	Conflicting_interpretations_of_pathogenicity
chr2_21005467_A_T	rs12713540	237734	APOB	missense_variant	Conflicting_interpretations_of_pathogenicity
chr2_21005858_C_A	rs148127628	627682	APOB	missense_variant	Uncertain_significance
chr2_21006160_G_A	rs201736972	431988	APOB	missense_variant	Uncertain_significance
chr2_21006288_C_T	rs5742904	17890	APOB	missense_variant	Conflicting_interpretations_of_pathogenicity
chr2_21006360_G_A	rs375284245	548033	APOB	missense_variant	Uncertain_significance
chr2_21006737_C_T	rs1799812	265890	APOB	synonymous_variant	Conflicting_interpretations_of_pathogenicity
chr2_21006985_A_G	rs186299244	334108	APOB	missense_variant	Conflicting_interpretations_of_pathogenicity
chr2_21007033_T_C	rs12720854	218452	APOB	missense_variant	Conflicting_interpretations_of_pathogenicity
chr2_21007574_G_A	rs145777339	334114	APOB	synonymous_variant	Conflicting_interpretations_of_pathogenicity

chr2_21008406_G_A	rs72653095	218451	APOB	missense_variant	Conflicting_interpretations_of_pathogenicity
chr2_21008515_T_G	rs2163204	218450	APOB	missense_variant	Conflicting_interpretations_of_pathogenicity
chr2_21008720_G_A	rs6413458	237751	APOB	synonymous_variant	Conflicting_interpretations_of_pathogenicity
chr2_21008734_G_A	rs372245645	630608	APOB	missense_variant	Uncertain_significance
chr2_21009172_C_T	rs1801696	189300	APOB	missense_variant	Conflicting_interpretations_of_pathogenicity
chr2_21009253_C_T	rs148170480	334125	APOB	missense_variant	Conflicting_interpretations_of_pathogenicity
chr2_21009583_A_T	rs72653092	218448	APOB	missense_variant	Conflicting_interpretations_of_pathogenicity
chr2_21010732_C_T	rs72653084	237748	APOB	missense_variant	Conflicting_interpretations_of_pathogenicity
chr2_21011005_C_T	rs368970025	548062	APOB	missense_variant	Conflicting_interpretations_of_pathogenicity
chr2_21011802_C_T	rs151009667	189304	APOB	missense_variant	Conflicting_interpretations_of_pathogenicity
chr2_21012043_A_G	rs72653083	477809	APOB	synonymous_variant	Conflicting_interpretations_of_pathogenicity
chr2_21015099_G_A	rs148959244	252482	APOB	missense_variant	Uncertain_significance
chr2_21015451_G_A	rs72653077	237744	APOB	missense_variant	Conflicting_interpretations_of_pathogenicity

chr2_21015495_C_T	rs12713843	281142	APOB	missense_variant	Conflicting_interpretations_of_pathogenicity
chr2_21015541_C_G	rs12713844	218443	APOB	missense_variant	Conflicting_interpretations_of_pathogenicity
chr2_21016655_C_T	rs72653071	255982	APOB	splice_region_variant,splice_polypyrimidine_tract_variant,intron_variant	Conflicting_interpretations_of_pathogenicity
chr2_21019869_C_T	rs151193347	334163	APOB	synonymous_variant	Conflicting_interpretations_of_pathogenicity
chr2_21022941_G_A	rs1801700	255981	APOB	synonymous_variant	Conflicting_interpretations_of_pathogenicity
chr2_21023017_G_A	rs12714097	490389	APOB	missense_variant	Conflicting_interpretations_of_pathogenicity
chr2_21026844_C_T	rs12691202	128421	APOB	missense_variant	Conflicting_interpretations_of_pathogenicity
chr2_21026872_G_A	rs756184175	334170	APOB	synonymous_variant	Conflicting_interpretations_of_pathogenicity
chr2_21029662_G_A	rs13306194	255979	APOB	missense_variant	Conflicting_interpretations_of_pathogenicity
chr2_21032294_G_T	rs12714224	265879	APOB	intron_variant	Uncertain_significance
chr2_21032420_A_C	rs766709743	925433	APOB	missense_variant	Uncertain_significance
chr2_21033509_T_C	rs199937544	627784	APOB	missense_variant	Conflicting_interpretations_of_pathogenicity

chr2_21033533_G_C	rs72653061	265876	APOB	splice_polypyrimidine_tract_variant,intron_variant	Conflicting_interpretations_of_pathogenicity
chr2_21033534_T_G	rs12720810	265877	APOB	splice_polypyrimidine_tract_variant,intron_variant	Conflicting_interpretations_of_pathogenicity
chr2_21043911_A_G	rs758450840	897863	APOB	missense_variant	Uncertain_significance
chr2_21044060_G_C	rs1800480	265871	APOB	5_prime_UTR_variant	Conflicting_interpretations_of_pathogenicity
